# Magnetic resonance imaging indicators for neurological outcome after surgery in patients with intramedullary spinal ependymomas

**DOI:** 10.1097/MD.0000000000028682

**Published:** 2022-01-28

**Authors:** Yongqiang Ma, Bofeng Bai, Xihai Zhao, Lixue Wang, Benqi Zhao, Yi Guo, Hongfang Yin, Xiaofei Zhang, Zhuozhao Zheng

**Affiliations:** a Department of Radiology, Beijing Tsinghua Changgung Hospital, School of Clinical Medicine, Tsinghua University, Beijing, China; b Center for Biomedical Imaging Research, Department of Biomedical Engineering, Tsinghua University School of Medicine, Beijing, China; c Department of Neurosurgery, Beijing Tsinghua Changgung Hospital, School of Clinical Medicine, Tsinghua University, Beijing, China; d Department of Pathology, Beijing Tsinghua Changgung Hospital, School of Clinical Medicine, Tsinghua University, Beijing, China; e Department of Clinical Epidemiology and Biostatistics, Beijing Tsinghua Changgung Hospital, School of Clinical Medicine, Tsinghua University, Beijing, China.

**Keywords:** ependymoma, intramedullary tumors, magnetic resonance imaging, neurological outcome, surgical resection

## Abstract

This is a retrospective study. The aim of this study was to determine the indicators of neurological outcome after surgery in patients with intramedullary spinal ependymomas by using magnetic resonance imaging (MRI).

A total of 106 consecutive patients (mean age: 42.4 ± 1.3 years; 52.8% male) diagnosed with intramedullary spinal ependymomas were retrospectively recruited. All patients underwent spine MRI and subsequent surgical resection for the spinal tumors. Data regarding clinical symptoms and pathological grades of tumors were collected from clinical records. The McCormick score was used for grading patients’ neurological status before and after surgery at 12 months. Good outcome was defined as stable McCormick score (McC) score (no change of McC score between preoperation and post-operation at 12 months) or improvement in McC score (post-operative McC score at 12 months < preoperative McC score). Poor outcome was determined when there was an increase in McC score at 12 months after surgery. The MRI characteristics of spinal ependymomas between patients with good and poor neurological outcomes were compared. Logistic regression was performed to assess the association between MRI characteristics of tumors and post-operative neurological outcomes.

Patients with poor neurological outcomes had larger longitudinal length (4.7 ± 0.5 vs 3.3 ± 0.2, *P* = .004) and higher enhancement signal-to-noise-ratio (SNR) (102.4 ± 12.3 vs 72.8 ± 4.6, *P* = .022) than those with good neurological outcomes. After adjusting for confounding factors, longitudinal length (OR, 0.768; 95% CI, 0.604–0.976; *P* = .031) and enhancement SNR (OR, 0.988; 95% CI, 0.978–0.999; *P* = .026) of spinal ependymomas were significantly associated with poor neurological prognosis.

The longitudinal length of tumor and enhancement SNR on T1-weighted images are independently associated with neurological outcome after surgery.

## 1 Introduction

Intramedullary spinal cord tumors (IMSCT) are very rare, accounting for only 2% to 4% of all central nervous system (CNS) tumors.^[1]^ Of these, ependymomas are the most commonly encountered tumors.^[2]^ Currently, surgical resection is the mainstream treatment modality for spinal ependymomas. The goal of surgery is to maintain or improve neurological function and ensure maximal safe resection of the tumor. A substantial number of retrospective studies on intramedullary spinal ependymomas in the last decade have reported that various factors may be predictive for neurological outcomes after surgery, including age, sex, duration of symptoms before surgery, preoperative neurological status, extent of tumor removal, pathological World Health Organization (WHO) grade, tumor size, tumor location, and tumor margins.^[3–10]^ However, most existing studies have smaller sample size and the evidence on the role of the above-mentioned influencing factors in neurological outcomes is controversial. Magnetic resonance imaging (MRI) is now recognized as an ideal modality for assessing the characteristics of spinal ependymomas in clinical settings, because MRI provides multidimensional and multi-contrast information of tumors. However, a comprehensive and quantitative evaluation of the MRI features of intramedullary spinal ependymomas and their association with postoperative neurological outcomes is still lacking. The purpose of this retrospective study was to determine the association between MRI characteristics of intramedullary spinal ependymomas at baseline and the 1-year neurological outcome after surgery.

## 2 Methods and materials

### 2.1 Study population

Patients who underwent surgery for spinal ependymomas and MRI before the surgery between January 2015 and June 2018 were retrospectively recruited. Patients with myxopapillary ependymomas were excluded in this study, because these tumors are located in the filum terminalis and, by definition, are not intramedullary tumors. Patients who failed to be followed-up for at least 12 months or had contraindications to MRI examination were excluded. The clinical information including age, sex, duration of clinical symptoms, and tumor grades determined by pathology was collected from medical records. All patients presenting with neurological clinical features were evaluated thoroughly by academic residents of neurosurgery. The McCormick (McC) scale was used to grade patients’ neurological status before surgery (see Supplemental Digital Content Table which illustrates the criteria for McCormick classification).^[2]^ The study protocol was approved by the institutional review board, and written consent form was obtained for each patient.

### 2.2 Operative and perioperative course

All patients underwent their first operation at our institution. The operation followed the usual procedures for intramedullary spinal cord tumors.^[11]^ Intraoperative neurophysiological monitoring (IOM) was regularly used to monitor all cases.^[12]^ An operating microscope was routinely used in all operations. There was no peri- or intraoperative radio- or chemotherapy used. The excised tumor mass was then examined by 2 senior pathologists concurrently to arrive at a histological diagnosis.

### 2.3 MR imaging at baseline

The MRI was performed on a 3.0T MR scanner (Discovery 750, GE Healthcare, Milwaukee, USA) with a standard 8-channel spine coil. For MRI, the following imaging sequences were acquired: fast spin-echo (FSE) T1-weighted (T1W) imaging (sagittal acquisition), FSE T2-weighted (T2W) imaging (sagittal acquisition), and FSE T2W imaging with fat saturation (axial acquisition) sequences. The imaging parameters were as follows: T1W imaging (sagittal), repeat time (TR)/echo time (TE), 600 ms/14 ms; T2W imaging (sagittal), TR/TE, 2980 ms/101 ms; and T2W imaging with fat saturation (axial), TR/TE, 6075 ms/99 ms. Further, the field of view, slice thickness, and matrix were 30 cm × 30 cm, 4 mm, and 352 × 256 for sagittal acquisition, respectively, and were 20 cm × 20 cm, 5 mm, and 320 × 224, respectively, for axial acquisition. After intravenous administration of gadolinium diethylene triamine pentaacetic acid (Gd-DTPA) (Omniscan, GE Healthcare) at a dose of 0.1 mmol/kg, axial, sagittal, and coronal FSE T1W imaging (TR/TE 600 ms/14 ms) was acquired with an field of view of 30 cm × 30 cm, matrix of 352 × 256, and slice thickness of 4 mm.

### 2.4 MR image analysis

Two experienced neuroradiologists (M.Y., Z.Z.) with >5 years’ experience in neuroimaging and blinded to the histopathological results independently evaluated the MR images with consensus. During MR image analysis, the following features were analyzed using the picture archiving and communication system (PACS) workstation:

1. longitudinal length of the tumor which was defined as the number of vertebrae that the tumor covers;
2. Signal-to-noise-ratio (SNR) on T2WI which was defined as the ratio of the signal of tumor at the region with homogenous intensity to the noise of air;
3. enhancement patterns which were categorized into no enhancement, homogenous enhancement, and inhomogeneous enhancement;
4. enhancement SNR which was defined as the ratio of the signal of tumor at the region with enhancement to the noise of air;
5. clearness of tumor margin;
6. presence of cyst;
7. presence of hemorrhage;
8. presence of peripheral edema; and
9. tumor location.

The clearness of tumor margin was determined according to the boundaries between the tumor and surrounding spinal cord. The presence of hemorrhage was defined as hyperintense within the tumor on T1W imaging with fat saturation (signal intensity >1.5 times that of the adjacent spinal cord). Peripheral edema was defined as presence of hyperintense signal surrounding the lesions on T2WI without enhancement on T1WI.

### 2.5 Clinical follow-up

All patients underwent MRI preoperatively, 48 hours postoperatively, and then every 6 months until the last follow-up. The extent of the excision was graded into: gross-total resection (GTR) (no residual enhancement on initial postoperative MRI) and subtotal resection (STR) (<20% residual enhancement on initial postoperative MRI). The neurological status of all patients was also evaluated at discharge, 6 months, and 12 months after surgery. The neurological outcomes were classified into “good” and “poor” categories. Good outcome was defined as stable McCormick score (McC) score (the McC score does not change between pre- and post-operation at 12 months) or improvement in McC score (postoperative McC score at 12 months < preoperative McC score). Poor outcome was determined when there was an increase in McC score at 12 months after surgery.

### 2.6 Statistical analysis

Statistical Package for the Social Sciences version 11.0 (SPSS, Chicago, IL) and STATA 14.0 were used for statistical analysis. Means and standard deviation were calculated for continuous data with normal distribution. If the continuous variables showed non-normal distribution after normality test, the nonparametric Wilcoxon rank-sum test was used for the median comparison between the 2 groups. Median was also calculated for continuous data with non-normal distribution. Percentages and proportions were calculated for categorical data. Chi-Squared test was used to determine the differences in categorical variables between patients with good and poor neurological outcome. Univariate and multivariate logistic regressions were performed to assess the correlation between MRI characteristics of the tumor and postoperative neurological outcomes before and after adjusting for confounding factors. For all statistical tests, *P* < .05 was considered to indicate statistically significant difference.

## 3 Results

The mean age of the 106 recruited patients was 42.4 ± 1.3 years and 56 (52.8%) patients were male. After surgery, GTR was achieved in 101 (95.3%) patients, and STR was achieved in 5 (4.7%) patients. Pathological results after surgery revealed that 102 (96.2%) of the 106 patients had grade II ependymoma and 4 (3.8%) had Grade III anaplastic ependymomas. The mean follow-up time was 28.3 ± 35.5 months. No patients were lost to follow-up. All 4 Grade III patients had undergone GTR, and 3 (75.0%) of them had postoperative recurrence. Of the 102 Grade II patients, 97 (95.1%) had undergone GTR and 5 (4.9%) patients showed postoperative recurrence.

### 3.1 McC score

In this study population, the preoperative symptoms included sensory disturbance (n = 101, 95.3%); motor weakness (n = 35, 33.0%), sphincter dysfunction (n = 17, 16.0%), and gait ataxia (n = 26, 24.5%). The median duration of symptoms before diagnosis was 29.5 ± 3.6 months. The analysis of preoperative McC score showed that 68 patients (64.2%) were categorized as grade I, 30 (28.3%) as grade II, 5 (4.7%) as grade III, and 3 (2.8%) as grade IV. Twelve months after surgery, an improvement in neurological status was observed in 22 (20.8%) patients, maintained neurological status was observed in 62 (58.5%), and worsened status in 22 (20.8%).

### 3.2 MRI characteristics

Table 1 summarizes the clinical and MRI characteristics of the study population. Of the 106 ependymomas, 72 (67.9%), 23 (21.7%), and 11 (10.4%) were dominantly located in the cervical region, thoracic region, and lumbar region, respectively. The mean length of tumor involvement along the neuraxis was 3.6 spinal segments. On precontrast enhanced T1W images, 85 lesions (80.2%) showed isointense, 17 lesions (16.4%) showed slightly hypointense, and 4 lesions (3.8%) showed slightly hyperintense signals, respectively. All tumors appeared hyperintense on T2W images. Of all the tumors, 61 (57.5%) showed surrounding cord edema, 35 (33.0%) had hemorrhage, 91 (85.8%) had clear boundary on gadolinium-enhanced T1W images, 54 (50.9%) had central cysts, 57 (53.8%) had satellite cysts, and 19 (17.9%) had a “cap sign” at the poles of the tumor on T2W images, which is characterized by a rim of extreme hypointensity due to hemosiderin. On gadolinium-enhanced T1W images, 104 (98.1%) tumors showed contrast enhancement.

**Table 1:** Clinical and MR imaging characteristics of study population (n = 106).

	Mean ± SD and Median or n (%)			
	All patients (n = 106)	Good prognosis (n = 84)	Poor prognosis (n = 22)	*P* value
Age, yr	42.4 ± 1.3	43.7 ± 1.4	37.4 ± 2.6	.045
Sex, male	56 (52.8)	44 (52.4)	12 (54.5)	.856
Duration of symptoms, months	29.5 ± 3.6	27.4 ± 3.3	37.4 ± 12.0	
Median	13.5	15.5	12	.478^†^
Tumor location				
Cervical	72 (67.9)	60 (71.4)	12 (54.5)	.252
Thoracic	23 (21.7)	17 (20.3)	6 (27.3)	
Lumbar	11 (10.4)	7 (8.3)	4 (18.2)	
MR imaging characteristics of tumor				
longitudinal length^∗^	3.6 ± 0.2	3.3 ± 0.2	4.7 ± 0.5	
Median	3	3	4.5	.004^†^
SNR on T2WI	32.2 ± 1.6	31.0 ± 1.6	36.6 ± 4.6	
Median	29.09	29.09	29.88	.505^†^
Enhancement patterns				
No enhancement	2 (1.9)	1 (1.2)	1 (4.6)	.572
Homogenous enhancement	37 (34.9)	30 (35.7)	7 (31.8)	
Heterogeneous enhancement	67 (63.2)	53 (63.1)	14 (63.6)	
Enhancement SNR	79.0 ± 4.6	72.8 ± 4.6	102.4 ± 12.3	
Median	63.2	60.6	97.6	.022^†^
Margins				
Clear	91 (85.8)	74 (88.1)	16 (72.7)	.261
Unclear	15 (14.2)	10 (11.9)	6 (27.3)	
Presence of cysts				
Central cyst	54 (50.9)	45 (53.6)	9 (40.9)	.290
Satellite cyst	57 (53.8)	46 (54.8)	11 (50.0)	.690
Presence of hemorrhage	35 (33.0)	29 (34.5)	6 (27.3)	.520
Peripheral edema	61 (57.5)	50 (59.5)	11 (50.0)	.421
Extent of resection				
Gross-total resection	101 (95.3)	80 (95.2)	21 (95.5)	.966
Subtotal resection	5 (4.7)	4 (4.8)	1 (4.5)	
Tumor grade				<.001
Ependymoma (E II)	102 (96.2)	84 (100.0)	18 (81.8)	
Anaplastic ependymoma (AE III)	4 (3.8)	0 (0.0)	4 (18.2)	
Tumor recurrence	9 (8.5)	5 (6.0)	4 (18.2)	.067
Preoperative McC score				
I	68 (64.2)	58 (69.0)	10 (45.5)	.007
II	30 (28.3)	20 (23.8)	10 (45.5)	
III	5 (4.7)	5 (6.0)	0 (0.0)	
IV	3 (2.8)	1 (1.2)	2 (9.0)	

∗ Longitudinal length of tumorwas measured by the number of vertebras that the tumor covers.

† Wilcoxon rank-sum test was used because of non-normal distribution for Duration of symptoms, longitudinal length, SNR on T2WI, and Enhancement SNR.

### 3.3 Association between MRI characteristics and neurological outcome

**Figure 1:**
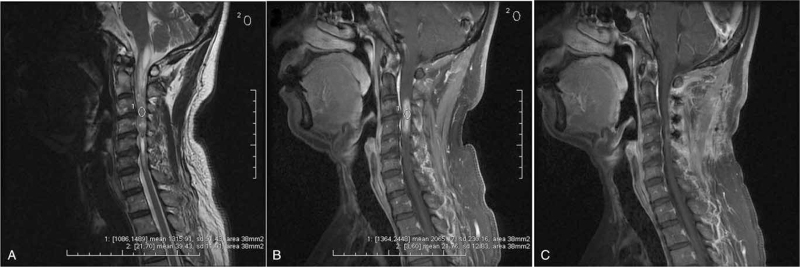
Example for ependymoma with high enhancement SNR and poor functional outcome (preoperative McC scale II vs postoperative McC scale III) after surgery in a 55-year-old male patient. Preoperative sagittal T2W images (A) demonstrated an intramedullary mass at the level of C3 to C4 (longitudinal length = 2 vertebral segments) with satellite cysts at both the rostral and caudal ends of the tumor. The tumor was slightly hyperintense on T2W images. The region of interest (ROIs) of the sagittal T2W images were taken from a 38 mm^2^ region (small circle) in the lesion area. The SI value was 1315.91. The ROI of the reference region was obtained from a 38 mm^2^ region (same small circle) in the area of surrounding air; its SI value was 39.43. The SNR on the T2W images was 33.37 (1315.91/39.43). Contrast-enhanced sagittal T1W images (B) showed strong and homogeneous enhancement with clear tumor margins. The ROIs were taken from a 38 mm^2^ region (small circle) on the area with enhancement; its SI value was 2065.17. The ROIs of the reference region were obtained from a 38 mm^2^ region (same small circle) in the area of surrounding air; the SI value was 21.76. The SNR on the contrast-enhanced T1W images was 94.91 (2065.17/21.76). Postoperative contrast-enhanced T1W images (C) demonstrated complete resection of the lesion. The histological result was ependymoma (E II). ROI = region of interest, SI = signal intensity, SNR = signal-to-noise-ratio, T1W = T1 weighted, T2W = T2 weighted.

**Figure 2:**
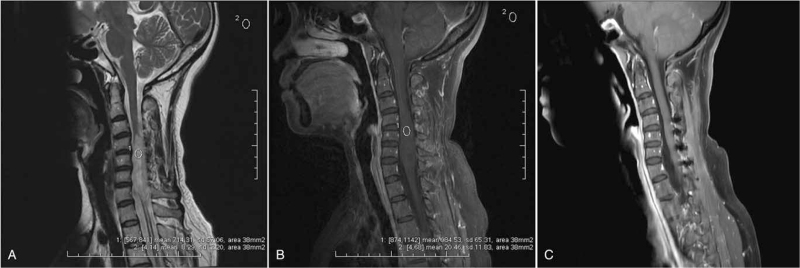
Example for ependymoma with low-enhancement SNR and good functional outcome (preoperative McC scale I vs. postoperative McC scale I) after surgery in a 43year-old female patient. Preoperative sagittal T2W images (A) demonstrated that the cord was stretched from C4 to C7 (longitudinal length = 4 vertebral segments) by a uniform mass lesion with syringomyelus at the caudal ends of the tumor. The tumor was heterogeneously hyperintense on T2W images. The SNR on the T2W images was 86.17 (714.31/8.29). Contrast-enhanced sagittal T1W images (B) showed slightly and inhomogeneous enhancement with unclear tumor margins. The SNR on the contrast enhanced T1W images was 48.12 (984.53/20.46). Postoperative contrast-enhanced T1W images (C) demonstrated complete resection of the lesion. The histological result was ependymoma (E II). ROI = region of interest, SI = signal intensity, SNR = signal-to-noise-ratio, T1W = T1 weighted, T2W = T2 weighted.

**Figure 3:**
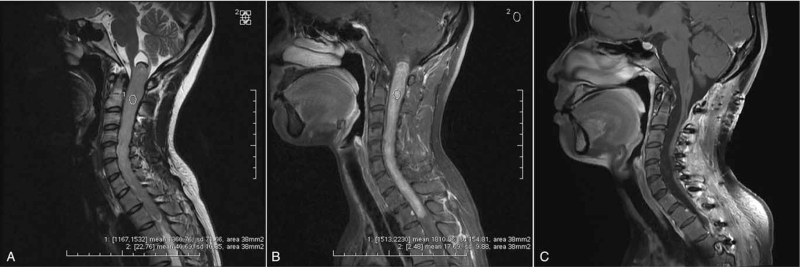
Example of a long ependymoma showing high enhancement SNR and poor functional outcome (preoperative McC scale II vs postoperative McC scale III) after surgery in a 28-year-old female patient. Preoperative sagittal T2W images (A) demonstrated isointense expansion of the spinal cord from the medulla oblongata to T2 (longitudinal length = 9 vertebral segments) with satellite cyst at both the rostral and caudal ends of the tumor. There was a “cap sign” at the rostral end of the tumor. The SNR on the T2W images was 33.44 (1360.76/40.69). Preoperative T1W images with contrast enhancement (B) showed homogeneous tumor enhancement with clear tumor margins. The SNR on the contrast-enhanced T1W images was 102.37 (1810.86/17.69). Postoperative T1W images with contrast enhancement (C) demonstrated complete resection of the lesion and myelatrophy. The histological result was ependymoma (E II). ROI = region of interest, SI = signal intensity, SNR = signal-to-noise-ratio, T1W = T1 weighted, T2W = T2 weighted.

The results on the association between tumor characteristics and neurological outcome are detailed in Table 2. Univariate logistic regression analysis showed that tumor length (OR, 0.735; 95% CI, 0.589–0.919; *P* = .007) and enhancement SNR (OR, 0.988; 95% CI, 0.979–0.997; *P* = .012) were significantly associated with neurological outcome. After adjusting for age, these associations, namely tumor length (OR: 0.774, 95% CI: 0.613–0.977, *P* = .031) and enhancement SNR (OR: 0.989, 95% CI: 0.979–0.999, *P* = .026) remained statistically significant. After further adjusting for all confounding factors including age, other MRI features of tumor, tumor grade, and preoperative McC score, these associations (tumor length: OR = 0.768, 95% CI = 0.604–0.976, *P* = .031; enhancement SNR: OR = 0.988, 95% CI = 0.978–0.999, *P* = .026) still remained statistically significant. No significant association was found between all other factors with neurological outcome (all *P* > .05). Figure 1 depicts the imaging findings in a patient who had high enhancement SNR in an ependymoma and suffered from poor neurological outcome. Figure 2 showed the imaging features of a patient who had low enhancement SNR in an ependymoma and good neurological outcome. Figure 3 shows the imaging features of a patient who had a long ependymoma and high enhancement SNR and suffered from poor neurological outcome.

**Table 2:** Association between tumor characteristics and prognosis.

	Poor neurological prognosis								
	Univariate regression			Multivariate regression^∗^			Multivariate regression^†^		
	OR	95% CI	*P*	OR	95% CI	*P*	OR	95% CI	*P*
Tumor length	0.735	0.589–0.919	.007	0.774	0.613–0.977	.031	0.768	0.604–0.976	.031
Enhancement SNR	0.988	0.979–0.997	.012	0.989	0.979–0.999	.026	0.988	0.978–0.999	.026

∗ adjusted for age.

† adjusted for all other factors.

### 3.4 Association between preoperative McC score and neurological outcome

Of 84 patients with good neurological outcome, 58 (69.0%), 20 (23.8%), 5 (6.0%), and 1 (1.2%) had preoperative McC scores of 1, 2, 3, and 4, respectively. In contrast, of the 22 patients with poor neurological outcome, 10 (45.5%), 10 (45.5%), 0 (0%), and 2 (9.0%) had preoperative McC scores of 1, 2, 3, and 4, respectively. The 2 patients who had McC score of 4 before and after surgery were classified into the poor neurological outcome group because their neurological status deteriorated after surgery, and they died during the follow-up. The preoperative McC score between patients with good and poor neurological outcome was statistically significant (*P* = .007, Table 1).

## 4 Discussion

This study investigated the association between MRI characteristics of tumors and postoperative neurological outcomes in patients with intramedullary spinal ependymomas. We found that the tumor length and enhancement SNR on T1W images were significantly associated with neurological outcome after surgery, before and after adjusting for confounding factors. Our findings suggest that patients with larger length or higher enhancement SNR of intramedullary spinal ependymomas may have poor neurological outcome after surgery.

In the present study, patients with larger tumor length had poor outcome after surgery. This is in agreement with the study by Ebner et al, which also confirmed that patients with extended intramedullary lesions have a worse neurological status preoperatively, postoperatively and in the 3-month follow-up. In the study by Ebner et al, 46 patients were recruited and divided into group A (lesions fewer than 3 vertebral segments) and group B (lesions over 3 or more vertebral segments). Their results showed that patients in group B had significant lower preoperative McCormick and Klekamp-Samii grades than those in group A with *P* < .05.^[7]^ Peker et al analyzed 21 cases of intramedullary ependymomas and concluded that longer length was significantly associated with development of dysesthesia postsurgery.^[13]^ This may be because a large-sized tumor leading to rupture of the tumor capsule and subsequent infiltration of surrounding neural tissue, intraoperative traction, and detachment may result in new deficits after surgery; hence, once the normal plasticity of the spinal cord is impaired because of extrusion by a large tumor, surgery offers few advantages with regard to neurological improvement.^[14,15]^

On pre-contrast MRI, intramedullary spinal ependymomas usually show high signal intensity on T2W images. On contrast-enhanced T1W images, most tumors show homogeneous enhancement and often have clear margins.^[16]^ However, the MR signal intensity (SI) and degree of enhancement are variable owing to the different distributions of the vascular component of the connective tissue in the tumor mass.^[17]^ Chang et al found no significant association between MRI signal features and enhancement patterns and postoperative neurological outcome of intramedullary spinal ependymomas.^[18]^ However, quantitative evaluation of MR SI and enhancement degree of intramedullary spinal ependymoma in the evaluation of association with postoperative neurological outcomes is still lacking. In our study, we quantitatively evaluated MR SI on T2W images and contrast-enhancement degree on T1W images using SNR. We found that enhancement SNR in the poor neurological outcome group was significantly higher than that in the good neurological outcome group. Enhancement SNR is the ratio of the tumor signal at the region with enhancement to the noise of air, which reflects the degree of enhancement. Contrast enhancement of tumors reflects not only microvascular proliferation of the tumor lesion but also disrupted blood-brain barrier. ^[19,20]^ The possible mechanism is that intramedullary spinal ependymomas with higher degree enhancement may have more feeding branches of the anterior spinal cord artery for both the tumor and peripheral spinal cord. After these feeders were coagulated and severed, peripheral spinal cord ischemia may be more serious. Our results did not show significant difference in SNR on T2W images between the poor and good postoperative neurological outcome groups.

In the present study, patients with higher pre-operative McC score were more likely to have poor neurological outcomes after surgery, though preoperative McC score was not an independent indicator for neurological outcome. Our findings are consistent with literature reports.^[6,21,22]^ In the study by Chang et al, of 31 patients with spinal enpendymomas, 11 (35%) with preoperative Nurick's grade < 4 showed improvement in neurological status, whereas those with preoperative Nurick's grade = 4 showed poorer neurological status (*P* = .05). This study suggested that patients with worse preoperative neurological statuses had fewer opportunities for neurological improvement.^[18]^ Furthermore, early diagnosis and timely microsurgical management have been suggested to be vital for positive outcomes.^[10,18]^

Most reports acknowledge that high-grade intramedullary spinal cord tumors had higher rates of recurrence but no effect on neurological outcome.^[23]^ Our findings are consistent with literature reports. The ependymal tumors were classified into 3 types according to WHO criteria: grade I: subependymoma or myxopapillary ependymoma (MPE); grade II: ependymoma (E II); and grade III: anaplastic ependymoma (AE III).^[24]^ Myxopapillary ependymomas growth in the filum terminalis are extramedullary tumors and can be resected with less morbidity compared to truly intramedullary ependymomas. Patients with myxopapillary ependymomas were excluded in this study. Benefiting from the application of neurophysiological monitoring and microneurosurgical techniques in the surgery, in the present study, although GTR was achieved in all 4 patients with high-grade anaplastic ependymomas, 3 (75.0%) patients experienced recurrence because of malignancy of this type of tumor.

Previous studies have indicated that age, sex, duration of symptoms before surgery, tumor location, tumor margins, and the extent of tumor removal can be prognostic factors for neurological outcomes.^[3,5,9,10,14]^ In contrast, no significant association was found between all these factors and the neurological outcomes in our study. Previous investigators believe that tumor location and tumor margins may not be the independent prognostic factors that usually influence the extent of tumor removal. Most reports acknowledge that the extent of tumor removal was the most significant prognostic factor influencing the postoperative outcome. Clearly, complete surgical resection is the best choice of treatment with a favorable outcome. Nevertheless, neurological outcome was not significantly affected by the extent of tumor resection (*P* = .966) in the present study. This possible reason might be limited by the number of cases with STR (5 cases, 4.7%).

Our study has some limitations. First, this is a single-center study with a relatively small number of anaplastic ependymoma (WHO grade III) patients. Second, this is a non-randomized trial lacking a control group and hence, we were unable to compare the outcomes between patients who did or did not undergo operation.

## 5 Conclusion

The longitudinal length and enhancement SNR on T1WI of intramedullary spinal ependymomas are independently associated with neurological outcome after surgery. Our findings suggest that patients with larger length tumor or higher enhancement SNR of intramedullary spinal ependymomas may have poor neurological outcome after surgery than other patients.

## 6 Ethics statement

Study participants voluntarily agreed to participate in the study and provided written informed consent before enrollment. The study was approved by the Ethics Committee of Beijing Tsinghua Changgung Hospital. All procedures performed in studies involving human participants were in accordance with the ethical standards of the institutional and/or national research committee and with the 1964 Helsinki declaration and its later amendments or comparable ethical standards.

## Author contributions

**Data curation:** Bofeng Bai, Lixue Wang, Benqi Zhao.

**Formal analysis:** Yongqiang Ma, Xihai Zhao.

**Investigation:** Yongqiang Ma, Yi Guo, Hongfang Yin.

**Methodology:** Yongqiang Ma, Xihai Zhao, Xiaofei Zhang.

**Resources:** Zhuozhao Zheng.

**Supervision:** Zhuozhao Zheng.

**Writing – original draft:** Yongqiang Ma.

## Supplementary Material

Supplemental Digital Content
