# Supplementary material for: Magnetic resonance imaging indicators for neurological outcome after surgery in patients with intramedullary spinal ependymomas
Source: Medicine (Baltimore). 2022 Jan 28;101(4):e28682. doi: 10.1097/MD.0000000000028682 (PMC8797478; doi:10.1097/MD.0000000000028682)
Supplement: Supplemental Digital Content [file medi-101-e28682-s001.docx]

**Supplemental Table.** Supplemental Table that illustrates the criteria for McCormick classification.

**Supplemental Table. Criteria for McCormick classification**

| Grade | Definition |
| --- | --- |
| I | Neurologically normal; mild focal deficit insignificantly affecting the function of the involved limb; mild spasticity; normal gait |
| II | Presence of sensorimotor deficit affecting function of the involved limb; mild-to-moderate gait difficulty; severe pain or dysesthetic syndrome |
| III | More severe neurological deficit; requires a cane and/or brace for ambulation or maintains significant bilateral upper-extremity impairment; may or may not function independently |
| IV | Severe neurological deficit; requires wheelchair or a cane and/or brace with bilateral upper-extremity impairment; usually not independent |
